# Active and Passive Immunization of Pan-Fungal Vaccine NXT-2 Reduces Morbidity and Mortality in an Immunosuppressed Murine Model of *Candida auris* Systemic Infection

**DOI:** 10.3390/vaccines13101033

**Published:** 2025-10-07

**Authors:** Kwadwo O. Oworae, Emily Rayens, Taylor I. Chapman, Daniel A. Wychrij, Lizabeth Buzzelli, Whitney Rabacal, Karen A. Norris

**Affiliations:** 1 Center for Vaccines and Immunology, University of Georgia, Athens, GA 30602, USA; koworae@uga.edu (K.O.O.); emily.x.rayens@kp.org (E.R.); taylor.chapman25@uga.edu (T.I.C.); daniel.wychrij@uga.edu (D.A.W.); lizabeth.buzzelli@uga.edu (L.B.); whitney.rabacal@uga.edu (W.R.); 2 Department of Infectious Diseases, University of Georgia, Athens, GA 30602, USA; 3 Department of Research & Evaluation, Kaiser Permanente Southern California, Pasadena, CA 91101, USA

**Keywords:** *Candida auris*, pan-fungal vaccine, NXT-2, invasive candidiasis, passive transfer, immunosuppression

## Abstract

Background: *Candida auris* has emerged as a significant public health threat causing life-threatening systemic infections. Of particular concern is the frequency of multidrug resistance, high transmissibility, and persistence in the environment; thus, there is a need for novel strategies to prevent and treat this infection. We previously generated a “pan-fungal” vaccine candidate, NXT-2, which induces protective immunity against several invasive fungal infections. Methods: In this study, we investigated the efficacy of NXT-2 immunization against systemic *C. auris* infection in an immunosuppressed murine model and investigated the possible mechanisms by which NXT-2 protection is mediated in vitro. Results: Active immunization afforded significant improvement in survival and reduced morbidity in neutropenic mice challenged intravenously with *C. auris* compared to controls (48.4% vs. 13.8%). To assess humoral immunity in promoting protection, passive immunization with NXT-2-specific IgG to neutropenic mice prior to the challenge with *C. auris* resulted in significantly higher survival (42% vs. 0%) and low morbidity compared to controls. Sera from NXT-2-immunized animals inhibited biofilm formation and enhanced opsonophagocytic killing of multiple *C. auris* clades in vitro. Conclusions: These findings show that immunization with NXT-2 improves survival in *C. auris* infection and that NXT-2 antibodies promote antifungal activity in vitro and in vivo. These results extend the range of the pan-fungal NXT-2 vaccine to include protection against systemic *C. auris*-mediated infection and provide a rationale for the development of NXT-2 monoclonal antibodies for the treatment of *C. auris* infections.

## 1. Introduction

The frequency of invasive fungal infections (IFIs) has increased dramatically in the past decade, both in the US and globally [1,2,3]. Recent analyses estimate that globally over 6.5 million people suffer from IFIs each year, with overall and attributable annual deaths of 3.8 million and 2.6 million, respectively [1]. Based on analysis of US hospitalization data, most of the morbidity and mortality in the US, as well as the associated medical costs, are the results of infections by fungal organisms of three genera, *Aspergillus*, *Pneumocystis,* and *Candida* [2,3]. The increase in frequency of IFIs is likely due to the rise in at-risk populations, increased incidence of drug-resistant pathogens, and emerging new fungal pathogens, including *Candida auris* as an important cause of invasive candidiasis (IC) [4,5,6,7]. It is important to note that a recent taxonomic reclassification has reassigned this species to the genus *Candidozyma* and is now *Candidozyma auris* [8].

First identified in 2009, infections due to *C. auris* now constitute approximately 20% of IC cases, with a mortality rate of over 60% [9]. Healthy individuals may be colonized with *C. auris*, and unlike other *Candida* species, *C. auris* persists in the environment with remarkable resistance to standard sanitation procedures. The high mortality associated with *C. auris* infections is attributed to multiple factors, including delayed diagnoses, high transmissibility, poor immune clearance, and a high frequency of drug resistance to common antifungal drugs [6,7,10,11,12]. Immune control of *Candida* infections is typically mediated by phagocytes that recognize β-glucans on the fungal surface. However, *C. auris* shields these pathogen-associated molecular patterns with a dense mannan coat, preventing recognition and clearance [12]. This immune evasion emphasizes the appeal of antibody-based therapeutics, as opsonization could expose the pathogen to more effective immune recognition and killing [13]. Notably, approximately 90% of all *C. auris* isolates demonstrate resistance to fluconazole, 35% to amphotericin B, and 4% to echinocandins [14,15,16]. *C. auris* isolates can be categorized into clades emerging from different parts of the world—Clade I (South Asia), II (East Asia), III (South Africa), IV (South America), and potentially V (Iran)—each displaying distinct pathogenic traits [17]. Furthermore, antifungal resistance patterns vary significantly among the different clades of *C. auris*, making the pathogen a serious public health concern [18].

Despite the significant clinical burden of IFIs, there have been efforts to develop vaccines against *C. auris* [19,20,21] and other *Candida* spp. [22,23,24,25,26,27], but only one has been to clinical trials [28]. However, there are no approved fungal vaccines. To address the growing public health concern, we have focused on the development of a novel antifungal vaccine that induces protective immunity to multiple fungal pathogens [29]. Development of a “pan-fungal” vaccine is a clinically relevant strategy, as individuals at risk of IFIs are susceptible to multiple fungal infections [2,30,31]. Thus, a vaccine directed to a single pathogen may not be sufficient to protect against a spectrum of diseases. The lead candidate advanced by our laboratory, NXT-2, is a consensus sequence based on the sequence homology of the KEX1 90-mer peptides from *Aspergillus fumigatus*, *Pneumocystis jirovecii*, *Cryptococcus neoformans*, and *Candida albicans*. The KEX1 sequence is a highly conserved protein sub-unit originally from *Pneumocystis*, with an internal amino acid sequence related to the fungal kexin-like protein family [32], and is expressed on the surface of several major fungi [29]. We have reported that immunization with NXT-2 induces protective immunity against *Pneumocystis* infection in an immunocompromised NHP model, IPA, and systemic *C. albicans* infections in immunosuppressed murine models [29]. This protective immunity was shown to be comparable to species-specific KEX1 peptides in the same preclinical models [29,32,33].

Given the broad efficacy of the NXT-2 vaccine against multiple fungal pathogens in immunosuppressed animal models and the importance of *C. auris* as an emerging pathogen in vulnerable populations and healthcare settings, we sought to determine whether immunization with NXT-2 induced protective immunity against systemic *C. auris* infection in a highly susceptible, neutropenic murine model and to examine the role of vaccine-induced humoral immunity in protection.

## 2. Materials and Methods

### 2.1. Expression and Purification of C. auris KEX1 (CAu.KEX1) and NXT-2

CAu.KEX1 (accession number KND99220, residue number 304 to 393) cloned in pET28b+ (Novagen) in *E. coli* BL21 (DE3) was expressed using standard bacteria expression protocol with IPTG, as described in [29]. The recombinant protein was purified by affinity chromatography using TALON resin (Takara Bio USA, Inc., San Jose, CA, USA) per the manufacturer’s protocol. The purity of the protein was assessed via SDS-PAGE and Western blot analysis.

The NXT-2 recombinant protein was developed as described by Rayens et al. [29]. The recombinant protein was purified by affinity chromatography using TALON resin (Takara Bio USA, Inc., San Jose, CA, USA). The protein was then concentrated using a 3kDa Amicon ^®^ Ultra-15 Centricon (Merck & Co, Inc., Rahway, NJ, USA) following the manufacturer’s instructions before dialyzing in 1× phosphate buffer saline (PBS, pH 7.4) at 4 °C overnight. The purity of the protein was assessed via SDS-PAGE Western blot. The purified protein was tested for endotoxin using the Pierce^TM^ Chromogenic Endotoxin Quant Kit (ThermoScientific, Waltham, MA, USA) following the manufacturer’s protocol.

### 2.2. Western Blot Analysis

Western blot was used to analyze the purity of the recombinant proteins. Ten μg of purified CAu.KEX1 or NXT-2 protein was run on two 15% SDS-polyacrylamide gels under reducing conditions using Spectra™ Multicolor Broad Range Protein Ladder (ThermoFisher, Waltham, MA, USA). The gels were stained with Coomassie stain. The protein from the other gel was transferred onto a nitrocellulose membrane and blocked in 5% nonfat milk overnight. The blot was then incubated for 2 h with 6x-His tag monoclonal antibody, as well as mouse sera obtained prior to and following the *C. auris* challenge. The blots were washed with PBS supplemented with 0.05% Tween-20 (ThermoFisher, Waltham, MA, USA) and incubated for an hour with horseradish peroxidase-conjugated goat anti-mouse IgG (ThermoFisher, Waltham, MA, USA). The blots were washed as described above before being developed with West Pico PLUS Chemiluminescent Substrate (ThermoFisher, Waltham, MA, USA). Images were taken using the Gel Imager.

### 2.3. Animals

CD-1 mice (male and female) were obtained from Charles River Laboratories: 6–8-week-old mice were used for the vaccination experiment, and 10–12-week-old male mice were used for the passive transfer experiment.

### 2.4. Immunization and Evaluation of Immunogenicity of NXT-2 in CD-1 Mice

CD-1 mice were immunized subcutaneously at the base of the tail with either 50 μg NXT-2 prepared 1:1 with the water:squalene adjuvant TiterMax (Sigma–Aldrich, Inc., St. Louis, MO, USA) (NXT-2-immunized) or with sterile 1× PBS and TiterMax (sham-immunized) according to the manufacturer’s guidelines. The endotoxin level of the purified protein in the final vaccine formulation was measured to be 0.7 EU per dose. The animals were bled before (baseline) and after (28 days) NXT-2 vaccination. Blood was centrifuged at 10,000× *g* for 15 min at 20 °C and serum samples were stored at −80 °C until used for the in vitro assays described below.

NXT-2 antibody titers were determined before (baseline) and after (28 days) NXT-2 vaccination using ELISA assay, as previously described [29]. Briefly, microtiter plates (ThermoFisher, Waltham, MA, USA) were coated at 5 µg/mL of purified NXT-2 and incubated overnight at 4 °C. To determine the reciprocal endpoint titer (RET), sera from baseline and sham-immunized mice were initially diluted starting at 1:100, while NXT-2-immunized mice were initially diluted at 1:1000. All samples were then serially diluted 2-fold. Horseradish peroxidase conjugate-goat anti-mouse IgG, IgG1, or IgG2a (Southern Biotech, Birmingham, AL, USA) were used as detection antibodies. A TMB substrate (ThermoFisher, Waltham, MA, USA) was used to develop the plates. The reaction was stopped with 1 M sulfuric acid, and the plates were read at 450 nm on a plate reader.

### 2.5. Immunosuppression, C. auris Challenge, and Monitoring

To create a neutropenic model that mimics an at-risk population, the mice were immunosuppressed as previously described in an invasive candidiasis model [19,29]. The mice were immunosuppressed with 200 mg/kg of cyclophosphamide (Sigma–Aldrich, Inc., St. Louis, MO, USA) intraperitoneally and 250 mg/kg of cortisone 21-acetate (Sigma–Aldrich, Inc., St. Louis, MO, USA) subcutaneously 2 days before and 3 days after the *C. auris* challenge. Trimethoprim-sulfamethoxazole was added to the drinking water of the mice during the immunosuppression period to prevent opportunistic bacterial infections. The effect of the cyclophosphamide/cortisone immunosuppression strategy on neutropenia was confirmed in a pilot cohort by neutrophil count via Wright-stained blood smears prepared prior to and three days following immunosuppression. Compared to baseline samples, neutrophil depletion was over 95% following drug treatment, consistent with previously reported data using the same model [29].

*C. auris* (Clade I, CAU-09, CDC & FDA AR Isolate Bank, Atlanta, GA, USA) was streaked on a Yeast Extract–Peptone–Dextrose (YPD) agar plate and incubated at 30 °C for 48 h. A single colony was inoculated into 10 mL of YPD broth and incubated overnight at 30 °C in a shaker (225 rpm). The fungal cells were harvested before washing 3 times with 1× PBS at 524 × *g* for 5 min. The cells were counted on a hemocytometer and adjusted to an appropriate concentration. The mice were challenged intravenously via the tail vein at 2.5 × 10^6^ cells/g body weight in 100 µL solution. Male mice, with an average weight of 40 g, were challenged with 1 × 10^8^ cells/100 µL, and female mice, with an average weight of 30 g, were challenged with 7.5 × 10^7^ cells/100 µL. To ensure uniformity of inoculum dose, if inoculum leakage was observed during the injection or i.v. injection failure occurred, those animals were excluded from further study. Of the sham-immunized animals, 5 of 20 male mice and 6 of 20 female mice were excluded. In the NXT-2-immunized cohort, 3 out of 20 male mice and 6 out of 20 female mice were excluded. The final number of mice used was 31 for the NXT-2-immunized and 29 for the sham cohort. The mice were then monitored daily after the challenge for changes in temperature, weight, and physical appearance. Humane endpoints were defined as temperature less than 30 °C (hypothermia), weight loss greater than 20%, or moribund condition (unresponsive to stimuli or development of neurological symptoms).

### 2.6. Generation of NXT-2 Polyclonal Antibodies (pAbs) for Passive Transfer

For the generation of polyclonal NXT-2 antibodies for the passive transfer experiment, male CD-1 mice were immunized subcutaneously at the base of the tail with either 40 μg NXT-2a (a modified construct of NXT-2 that eliminated the histidine tag and linker) prepared 1:1 with the water:squalene TiterMax (Sigma–Aldrich, Inc., St. Louis, MO, USA) (NXT-2 polyclonal antibodies (pAbs) or with PBS and TiterMax (sham-pAbs) according to the manufacturer’s guidelines. Blood was collected before immunization and from euthanized mice 28 days following immunization and centrifuged at 10,000× *g* for 15 min to separate the sera. The sera were aliquoted and stored at −80 °C until used.

Polyclonal immunoglobulin G antibodies were purified from pooled mice sera (NXT-2a immunized and sham immunized) using the HiTrap Protein G column (Cytiva, Marlborough, MA, USA) following the manufacturer’s protocol. The eluted antibodies were concentrated using a 30 kDa Amicon ^®^ Ultra-15 Centricon (Sigma–Aldrich, Inc., St. Louis, MO, USA) following the manufacturer’s protocol. To perform buffer exchange, about 20 mL of 1× PBS was added to the concentrated antibodies in the Centricon and centrifuged down to 1 mL at 4000× *g*. This step was repeated 3 times. The concentrations of the antibodies were measured using a nanodrop. The purified antibodies were stored at −80 °C.

### 2.7. Passive Transfer of NXT-2 Antibodies and C. auris Challenge

For the passive transfer experiment, immunosuppression, *C. auris* challenge, and animal monitoring were performed as described above. The mice received 200 µg of either purified NXT-2 pAbs or sham pAbs administered intraperitoneally 2 h before and 3 days after the challenge with *C. auris.* To ensure uniformity of inoculum dose, if inoculum leakage was observed during the injection or i.v. injection failure occurred, those animals were excluded from further study. Ten male mice were used for each group. Three mice were excluded from the NXT-2 pAbs group and four mice were excluded from the sham pAbs group. For the passive transfer experiment, seven mice were included in the NXT-2 cohort and six in the sham.

### 2.8. Euthanasia

The mice vaccinated for pAb generation were euthanatized 28 days post-vaccination for blood collection. In vaccination and passive transfer studies, the mice reaching humane endpoints as well as all mice at the end of the survival studies were euthanized. Euthanasia was performed in accordance with the recommendations of the American Veterinary Medical Association (AVMA) Guidelines.

### 2.9. Biofilm Inhibition and Opsonophagocytic Killing (OPK) Assays

To determine the possible mechanisms by which NXT-2 protection is mediated in vitro, biofilm inhibition and OPK assays were performed. *C. auris* isolates—Clade I isolate CAU-09 (South Asia), Clade II isolate CAU-01 (East Asia), Clade III isolate CAU-03 (South African), and Clade IV isolate CAU-05 (South American) (from the CDC & FDA AR Isolate Bank)—were cultured and prepared as described above.

*C. auris* cells in 1× Yeast Nitrogen Base media (YNB supplemented with 0.5% glucose) were seeded in a 96-well plate in a volume of 95 μL per well containing 1 × 10^6^ cells. An aliquot of 5 μL of heat-inactivated sera (inactivated at 56 °C for 30 min) from NXT-2-immunized or sham-immunized mouse or NHP was added to each well and incubated at 37 °C in a humid environment for 24 h. The plate was then overturned and gently tapped on a paper towel in order not to disturb the biofilm formed. A volume of 100 μL of methoxy-nitrosulfophenyl-tetrazolium carboxanilide (XTT, Sigma–Aldrich, Inc, St. Louis, MO, USA) solution spiked with 0.0001% menadione was added to each well. The plate was then covered and incubated at 37 °C overnight. An aliquot of 80 μL of supernatant was transferred from each well into a new 96-well plate (Genesee Scientific, El Cajon, CA, USA) and read on a plate reader (Biotek Epoch 2, Thermofisher, Waltham, MA, USA) at 490 nm. The average OD_490_ values were reported for each group. Archival sera samples obtained from 4 NXT-2-immunized and 4 sham-immunized NHPs were used for the biofilm inhibition assay. Briefly, the NHPs were immunized with either 100 μg of NXT-2a with Alhydrogel (Invivogen, San Diego, CA, USA) or sterile 1× PBS with Alhydrogel and boosted 4 weeks after. Blood was taken 2 weeks following immunization and centrifuged at 3000× *g* for 10 min at 20 °C. Sera were aliquoted and stored at −80 °C until used for the in vitro assays described below.

To perform the OPK assay murine macrophage cell line, RAW 264.7 (ATCC) was cultured in RPMI (Gibco, Waltham, MA, USA) supplemented with 1% FBS (Corning, Corning, NY, USA) and 1% Pen-Strep (Gibco, Waltham, MA, USA) and activated with 1 ng/mL lipopolysaccharide from *E. coli* O26:B6 (Sigma-Aldrich, Inc, St. Louis, MO, USA) for 24 h prior to the experiment, as previously described [29]. The activated macrophages were harvested and adjusted to a concentration of 2 × 10^6^ cells/mL. *C. auris* cell suspension was seeded at 2 × 10^5^ CFU/well in a 96-well plate. An aliquot of 10 µL of heat-inactivated sera (inactivated at 56 °C for 30 min) pooled from all NXT-2-immunized mice or sham-immunized mice was added and incubated for 30 min at 37 °C. The activated macrophages were then added to each well (1:1 ratio with fungal cells) in a total of 200 μL. After 2 h of incubation at 37 °C with gentle shaking, the contents of each well were serially diluted (10-fold), and 10 µL of the dilutions were plated in triplicate and grown for 48 h at 37 °C on YPD agar plates. Colony-forming units (CFUs) were counted and percent killing was calculated using the following formula: 1 − [CFUs from sample with (mouse serum + fungi + macrophages)/average CFU in samples with (fungi + macrophage)]. The average percentage killing was reported for each group.

### 2.10. Statistical Analyses

Statistical analyses were performed using GraphPad Prism (GraphPad Software version 10.4). A *p* value of less than 0.05 was considered significant. Survival curves between the NXT-2 and sham cohorts were analyzed by the Mantel–Cox test. Average survival time, biofilm inhibition, and opsonophagocytic killing percentage were analyzed using a two-tailed Student’s T-test (for samples ≥10) or Mann–Whitney U test (for samples <10). Changes in weight were analyzed using repeated-measures mixed modeling.

## 3. Results

### 3.1. Immunogenicity of C. auris KEX1 (CAu.KEX1) During Infection and Cross-Reactivity with NXT-2 Antibodies

**Figure 1:**
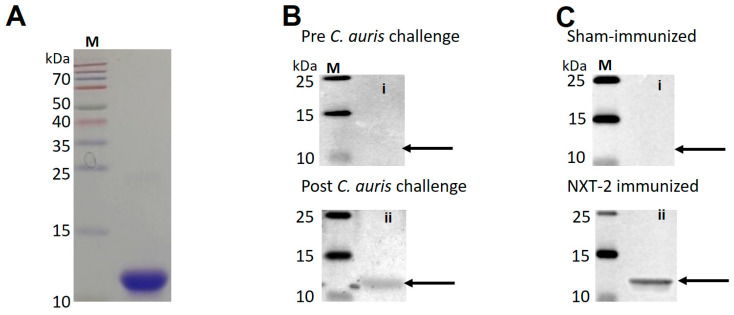
*Candida auris* recombinant antigen CAu.KEX1 is recognized by convalescent *C. auris* sera and anti-NXT-2 sera. Ten μg of purified CAu.KEX1 was run on for each gel. (**A**) CAu.KEX1 stained with Coomassie blue. (**B**) Western blots probed with sera from normal mice pre-*C. auris* infection (**top** panel, Bi) or post-*C. auris* infection (**bottom** panel, Bii). (**C**) Western blot of CAu.KEX1 probed with sera from sham-immunized mice (**top** panel, Ci) and NXT-2-immunized mice (**bottom** panel, Cii)). M = Molecular weight standards, arrows point to expected protein size.

Here, we demonstrated that antibodies to the *C. auris* KEX1 homologue CAu.KEX1 (Figure 1A) were likewise elicited during experimental systemic *C. auris* infection in neutropenic mice (Figure 1B). Additionally, anti-NXT-2 antibodies cross-react with the *C. auris* CAu.KEX1 (Figure 1C). These results provide the rationale for the evaluation of the protective efficacy of NXT-2 against *C. auris* infection in a neutropenic model.

### 3.2. NXT-2 Immunization Protects Mice Against Systemic C. auris Infection in Immunosuppressed Mice

**Figure 2:**
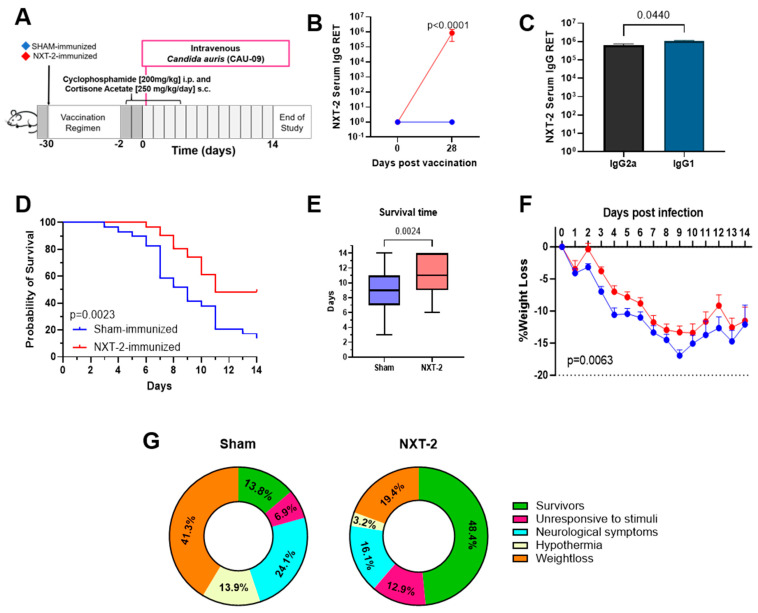
NXT-2 immunization improves survival in invasive candidiasis in the immunosuppressed mouse model. (**A**) Study design showing timeline of NXT-2 (red diamond) and sham immunizations (blue diamond), immunosuppression, challenge with *C. auris* (pink box)*,* and monitoring post-challenge. (**B**) Serum anti-NXT-2 IgG titers detected by ELISA before and after immunization between NXT-2- and sham-immunized mice. (**C**) IgG2a and IgG1 titers detected in NXT2-immunized mice. (**D**) Survival of mice immunized with NXT-2 (*n* = 15 of 31) was significantly increased compared to sham-immunized mice (*n* = 4 of 29) (Mantel–Cox test, *p* = 0.0023). (**E**) The mean survival time significantly increased in NXT-2-immunized mice compared to sham-immunized mice (T-Test, *p* = 0.0024). (**F**) NXT-2-immunizd mice had significantly less weight loss following *C. auris* challenge compared to the sham cohort. Data represents the mean ± SEM. Differences in weight loss were analyzed using repeated-measures mixed modeling (*p* = 0.0063)**.** (**G**) Symptomology of sham- and NXT-2-immunized mice throughout the course of the study.

To investigate the protective efficacy of the NXT-2 vaccine candidate against invasive *C. auris* infection, NXT2- or sham-immunized mice were immunosuppressed and intravenously challenged with *C. auris*. The study design is shown in Figure 2A. NXT-2 immunization elicited a robust antibody response with an RET of 8.47 × 10^5^ ± 1.16 × 10^5^ (Figure 2B), with a balanced IgG1 and IgG2a NXT-2-specific antibody response (Figure 2C). Following the intravenous challenge with *C. auris* in neutropenic mice, overall survival was higher in the NXT-2-immunized mice than the sham-immunized mice (48.4% (15 of 31) vs. 13.8% (4 of 29) (*p* = 0.0023), Figure 2D). When assessed based on sex, survival was higher in the NXT-2 immunized mice in both male mice (52.9% (7 of 17) vs. 20.0% (2 of 15), *p* = 0.0316) and female mice (42.9% (6 of 14) vs. 7.1% (1 of 14), *p* = 0.0416) compared to the sham-immunized mice (Supplementary Figure S1A,B). In both the NXT-2-immunized and sham-immunized mice, there was no significant difference in survival between the sexes (*p* = 0.6566 and *p* = 0.7652, respectively; Supplementary Figure S1C,D). The average survival time of the NXT-2-immunized mice was significantly longer (11.5 vs. 9.0 days, *p* = 0.0024, Figure 2E). The NXT-2-immunized mice had significantly less weight loss (as an indicator of morbidity) after *C. auris* infection compared to the sham-immunized mice over the course of the infection (*p* = 0.0063, Figure 2F). Of the sham-immunized mice that succumbed to *C. auris* infection, 41.3% (*n* = 12) experienced over 20% weight loss, 6.3% (*n* = 2) were unresponsive to stimuli, 13.9% (*n* = 4) experienced hypothermia, and 24.1% (*n* = 7) had neurological symptoms. In comparison, of the NXT-2-immunized mice that succumbed to infection, 19.4% (*n* = 6) experienced over 20% weight loss, 12.9% (*n* = 4) were unresponsive, 3.2% (*n* = 1) experienced hypothermia, and 16.1% (*n* = 5) had neurological symptoms (Figure 2G).

### 3.3. Passive Transfer of NXT-2 pAbs Confers Protection Against Systemic C. auris Infection in an Immunosuppressed Murine Model

**Figure 3:**
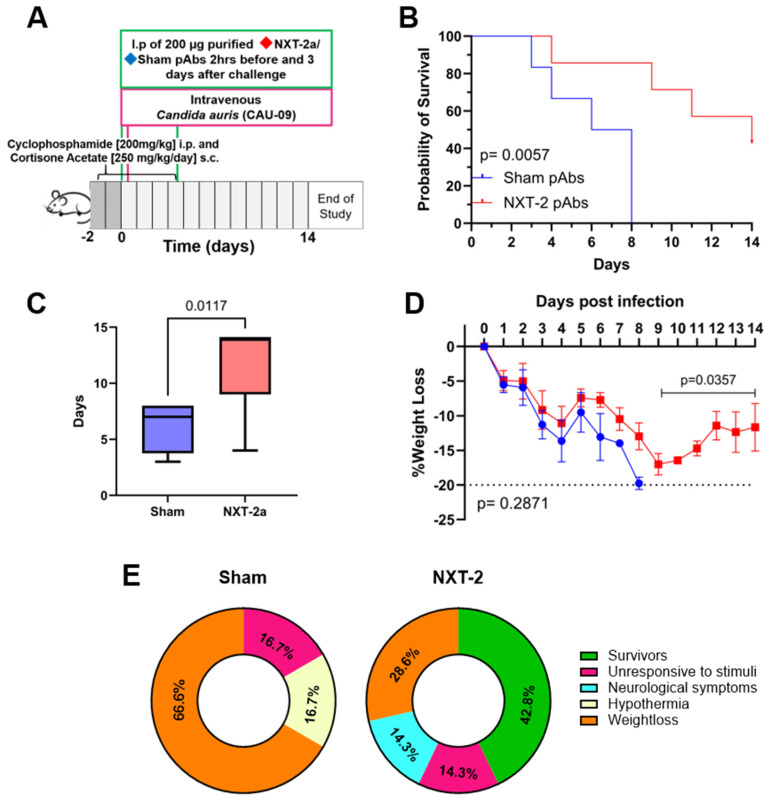
Passive transfer of NXT-2 pAbs confers protection against invasive candidiasis caused by *C. auris*. (**A**) Study design showing timeline of immunosuppression, pAbs administration (green box), *C. auris* challenge (pink box), and monitoring post-challenge. (**B**) Survival of mice receiving NXT-2 pAbs (*n* = 3 of 7) significantly increased compared to mice receiving sham pAbs (*n* = 0 of 6) (Mantel–Cox test, *p* = 0.0057). (**C**) The mean survival time of mice receiving NXT-2 pAbs significantly increased compared to mice receiving sham pAbs (Mann–Whitney test, *p* = 0.0117). (**D**) Weight loss following *C. auris* challenge was not significantly different in mice receiving NXT-2 pAbs compared to the sham cohort through Day 9 post-challenge. Data represents the mean ± SD. Differences in weight loss were analyzed using repeated-measures mixed modeling (*p* = 0.2871). Mice receiving NXT-2 pAbs had significant weight gain between Day 9 and Day 14 (Mann–Whitney test, *p* = 0.0357). (**E**) Symptomology of mice given sham and NXT-2 pAbs throughout the course of the study.

To determine the contribution of vaccine-induced NXT-2 antibodies in promoting immunity against invasive *C. auris* infection, purified NXT-2 pAbs were transferred to neutropenic mice 2 h before and 3 days after the intravenous challenge with *C. auris* (Figure 3A). Survival of the mice receiving NXT-2 pAbs treatment was significantly higher compared to the mice receiving pAbs from sham-immunized mice (42.8% (3 of 7) vs. 0% (0 of 6) (*p* = 0.0057, Figure 3B). Additionally, the mice that received NXT-2 pAbs had a significantly longer average survival time compared to the mice that received the sham pAbs (11.6 vs. 6.3 days, *p* = 0.0117, Figure 3C). There was no significant difference in weight between the NXT-2 pAbs-treated and sham-treated mice up to day 9 post-infection, when all untreated mice had succumbed to infection (Figure 3D, *p* = 0.2871). The mice in the NXT-2 pAb-treated group had a 5.8% increased weight from day 9 through the termination of the experiment (day 14, Figure 3D, *p* = 0.0357). At the end of the experiment, all mice that had received sham pAbs succumbed to *C. auris* infection, 66.6% (*n* = 4) experienced over 20% weight loss, 16.7% (*n* = 1) were unresponsive to stimuli, and 16.7% (*n* = 1) experienced hypothermia. In comparison, of the mice that received NXT-2 pAbs and that succumbed to infection, 28.6% (*n* = 2) experienced over 20% weight loss, 14.3% (*n* = 1) were unresponsive, and 14.3% (*n* = 1) had neurological symptoms (Figure 3E).

### 3.4. NXT-2 Sera Inhibit Biofilm Formation and Promote the Opsonophagocytic Killing (OPK) of Multiple C. auris Clades

**Figure 4:**
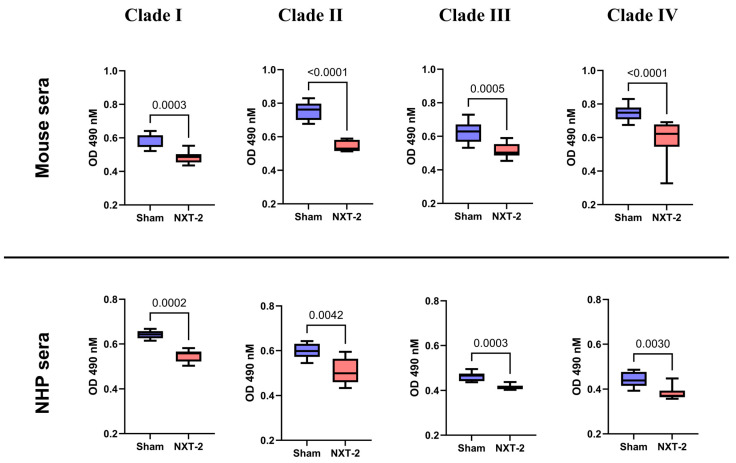
NXT-2 sera inhibit biofilm formation in multiple clades of *C. auris*. *C. auris* cells were incubated in the presence of heat-inactivated NXT-2 sera (red bar) or sham sera (blue bar) from mice and NHP. NXT-2 mice and NHP sera significantly reduced biofilm formation compared to the sham sera in the different *C. auris* clades (Mann–Whitney test). Clade I (CAU-09, South Asia), Clade II (CAU-01, East Asia), Clade III (CAU-03, South African), and Clade IV (CAU-05, South American).

To further examine the mechanisms of action of NXT-2 antibodies in protection against invasive *C. auris* infection, NXT-2 antibodies were tested for their ability to prevent biofilm formation and enhance the OPK of *C. auris*. NXT-2 hyperimmune mouse sera significantly reduced biofilm formation in all *C. auris* clades compared to the sham sera: Clade I (*p* = 0.0003), Clade II (*p* > 0.0001), Clade III (*p* = 0.0005), and Clade IV (*p* > 0.0001). To complement our findings with sera from NXT-2-vaccinated mice, we tested the ability of sera from NXT-2-immunized NHPs to inhibit biofilm formation across various *C. auris* clades. Similar results were observed for NXT-2 hyperimmune NHP sera, which significantly reduced biofilm formation compared to sham NHP sera in Clade I (*p* = 0.0002), Clade II (*p* = 0.0042), Clade III (*p* = 0.0003), and Clade IV (*p* = 0.0030) (Figure 4). The results with NHP antibodies support the cross-species relevance of the antifungal activity of NXT-2 vaccine-induced antibodies.

**Figure 5:**
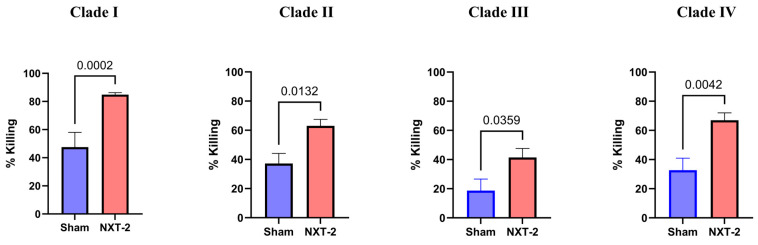
NXT-2 sera promote the opsonophagocytic killing of multiple clades of *C. auris*. The opsonophagocytic killing of murine macrophages was evaluated with sera from either NXT-2- or sham-immunized mice. Sera from NXT-2 sera-immunized mice (red bar) significantly enhanced the killing of *C. auris* compared to the sham (blue bar). The data is shown relative to the macrophage killing without sera. Statistical analyses were performed using the Mann–Whitney test. Clade I (CAU-09, South Asia), Clade II (CAU-01, East Asia), Clade III (CAU-03, South Africa), and Clade IV (CAU-05, South America).

The killing of *C. auris* by murine macrophages also significantly increased across all *C. auris* clades in the presence of NXT-2 hyperimmune sera compared to sham sera (Figure 5). For *C. auris* Clade 1, the percent killing was 85% with NXT-2 sera compared to 57% with the sham sera (*p* = 0.0002). The same trend was seen in the other clades, where percent killing was 65% compared to 48% (*p* = 0.0132), 41% compared to 19% (*p* = 0.0359), and 72% compared to 33% (*p* = 0.0042) for *C. auris* clades II, III, and IV, respectively. This represents a 17–39% increase in percent killing by the NXT-2 hyperimmune sera compared to the sham.

## 4. Discussion

The increasing frequency and clinical consequences of infections with pathogenic fungi present a critical emerging global health issue [1,2,3,34,35]. Hence, the WHO published the first ever Fungal Pathogen Priority report in 2022, placing multiple drug-resistant pathogens, such as *C. auris*, in the critical priority group [36]. To address these issues, we have explored a novel strategy to develop a single, antifungal vaccine capable of targeting multiple fungal pathogens in at-risk populations [29]. In the present study, we examined the protective efficacy of the ‘pan-fungal’ recombinant protein vaccine NXT-2 against IC caused by the multidrug-resistant *C. auris*. We first determined that, similar to other fungal KEX1 homologues [33,37,38], the *C. auris* KEX1 homologue induces an immunologic response during experimental *C. auris* infection, and that anti-NXT-2 antibodies are cross-reactive to the *C. auris* KEX1 antigen. The immunogenicity of the CAu.KEX1 during infections and the cross-reactivity of this protein with antibodies generated to the consensus protein NXT-2 provided the rationale for investigating NXT-2 as a vaccine candidate in an invasive *C. auris* infection model [32,33,37,38]. The key findings of this study are that both the active immunization of mice with NXT-2 and passive immunization with polyclonal NXT-2 antibodies significantly reduced morbidity and mortality in mice that were subsequently immunosuppressed and challenged systemically with a lethal dose of *C. auris*. A single vaccination with NXT-2 elicited a robust IgG response that was comparable to peak IgG titers from previous NXT-2 vaccine studies in mice and NHPs [29,32,33]. The murine model used in these studies is a well-established system that renders mice neutropenic and impairs the proliferation and function of other immune cells, thus increasing susceptibility to systemic *C. auris* infection [39]. Although this is an aggressive challenge model of systemic candidiasis infection in the context of profound immunosuppression, we observed a significant increase in overall survival, increased survival time, and reduced morbidity compared to sham-immunized animals.

In addition to the enhanced survival and reduced morbidity due to anti-NXT-2 IgG delivery in the passive transfer experiments, the role of anti-NXT-2 was evaluated in in vitro antifungal assays of biofilm inhibitions and enhanced phagocytosis. Biofilm formation is an important virulence factor of *Candida* spp. that protects the fungus from drug activity and immune clearance and promotes persistence and dissemination [11,40]. NXT-2 antibodies from immunized mice and NHPs were able to significantly inhibit biofilm formation of multiple clades of *C. auris* in vitro. Furthermore, anti-NXT-2 antibodies promoted the phagocytosis and killing of the fungus by murine macrophages compared to antibodies from sham-immunized mice. These findings demonstrate that NXT-2 antibodies improve the immune recognition and clearance of *C. auris*. Similar results observed with NHP sera provide valuable data supporting the evidence of cross-species immunogenicity of NXT-2 and antifungal functionality. These results are similar to the effect of anti-NXT-2 antibodies on *C. albicans* biofilm formation and opsonophagocytic killing, as previously reported [29], and support the conclusion that vaccine-induced anti-NXT-2 IgG antibodies are key effectors in the protective response.

*C. auris* exhibit clade-specific characteristics, including differences in metabolic processes, virulence factors, and resistance to antifungal drugs. These properties likely influence differences among clades regarding mortality, disease severity, and transmission efficiency [41,42,43,44]. For instance, Clade III isolates have a tendency toward increased aggregate formation, while Clade I isolates form pseudo-hyphae and are responsible for most outbreaks in the United States [42,44]. Clade IV has been shown to be highly pathogenic, while Clade II is the least pathogenic in experimental models of immunosuppressed mice [39]. The ability of NXT-2 antibodies to inhibit biofilm formation and enhance the opsonophagocytic killing of multiple clades of *C. auris* shows a broad range of antifungal activity and supports the conclusion that the critical immune target(s) of the vaccine is conserved across clades. These results support the concept that the immunologic range of the NXT-2 vaccine may include geographic areas with different clades or in individuals experiencing infection with multiple clades [45,46,47]. Additionally, we recently reported that NXT-2 immunization affords protection in a murine model of vulvovaginal candidiasis due to *Candida albicans* and that anti-NXT-2 antibodies promote antifungal activity [48]. Together, these results indicate that NXT-2 immunization affords protection against both systemic and localized *Candida* infections.

While these findings are promising, several important next steps remain. The cross-protective activity of NXT-2 antibodies against multiple *C. auris* clades was demonstrated in vitro and should be validated in vivo to fully establish their breadth and translational relevance. In addition, assessing the long-term durability of vaccine-induced immunity will be essential for understanding how NXT-2 can be used in at-risk populations. Addressing these questions will strengthen the case for NXT-2 as a pan-fungal vaccine candidate and also inform its potential combination with antifungal drug therapies to enhance protection and overcome resistance.

Together, the results presented here further support the concept of NXT-2 as a “pan-fungal” vaccine and provide pre-clinical evidence for further development of the NXT-2 vaccine candidate for multiple clinical indications. While active immunization with NXT-2 offers a path toward long-term protection in certain high-risk populations, it may not be feasible for individuals who are severely immunocompromised or acutely infected [49,50]. In such clinical situations, including individuals undergoing high-risk invasive or localized outbreaks of *C. auris* infections, the present study supports the concept of NXT-2-antibody-based therapeutics as a feasible alternative or additional strategy to standard antifungal treatment. As growing antifungal drug resistance becomes an urgent threat, these findings support the continued development of NXT-2 antibodies for the prevention and treatment of *C. auris* infection in these scenarios.

## 5. Conclusions

In summary, the emergence of *C. auris*, particularly multidrug-resistant isolates, is a significant public health threat, particularly in immunosuppressed individuals and in healthcare settings [51,52,53]. We demonstrate that active and passive immunization of NXT-2 provides protection against systemic infection. NXT-2-induced antibodies exhibited potent antifungal activity by inhibiting biofilm formation and promoting opsonophagocytic killing across multiple *C. auris* clades.

Future studies will focus on testing the long-term durability of the vaccine-induced immune response and its efficacy in different immunosuppression models to reflect various at-risk populations. Assessing the potential for synergy between the vaccine and standard antifungal treatments will also be essential for improving protection. Furthermore, investigating the vaccine in a transmission model will be key to understanding its potential for reducing the spread of *C. auris* in clinical and community settings [54]. Nonetheless, these findings highlight the potential of NXT-2 as a single, pan-fungal vaccine candidate and support the rationale for advancing both vaccine- and antibody-based therapeutic strategies to protect vulnerable populations from life-threatening fungal infections.

## Data Availability

The datasets used and analyzed during this study are available from the corresponding author on reasonable request.

## Supplementary Materials

The following supporting information can be downloaded at https://www.mdpi.com/article/10.3390/vaccines13101033/s1: Figure S1: Survival of male and female mice after *C. auris* challenge; Figure S2: Comparison of biofilm formation using mouse and NHP sera.
