# Supplementary material for: Active and Passive Immunization of Pan-Fungal Vaccine NXT-2 Reduces Morbidity and Mortality in an Immunosuppressed Murine Model of Candida auris Systemic Infection
Source: Vaccines (Basel). 2025 Oct 7;13(10):1033. doi: 10.3390/vaccines13101033 (PMC12567899; doi:10.3390/vaccines13101033)

Supplementary figures

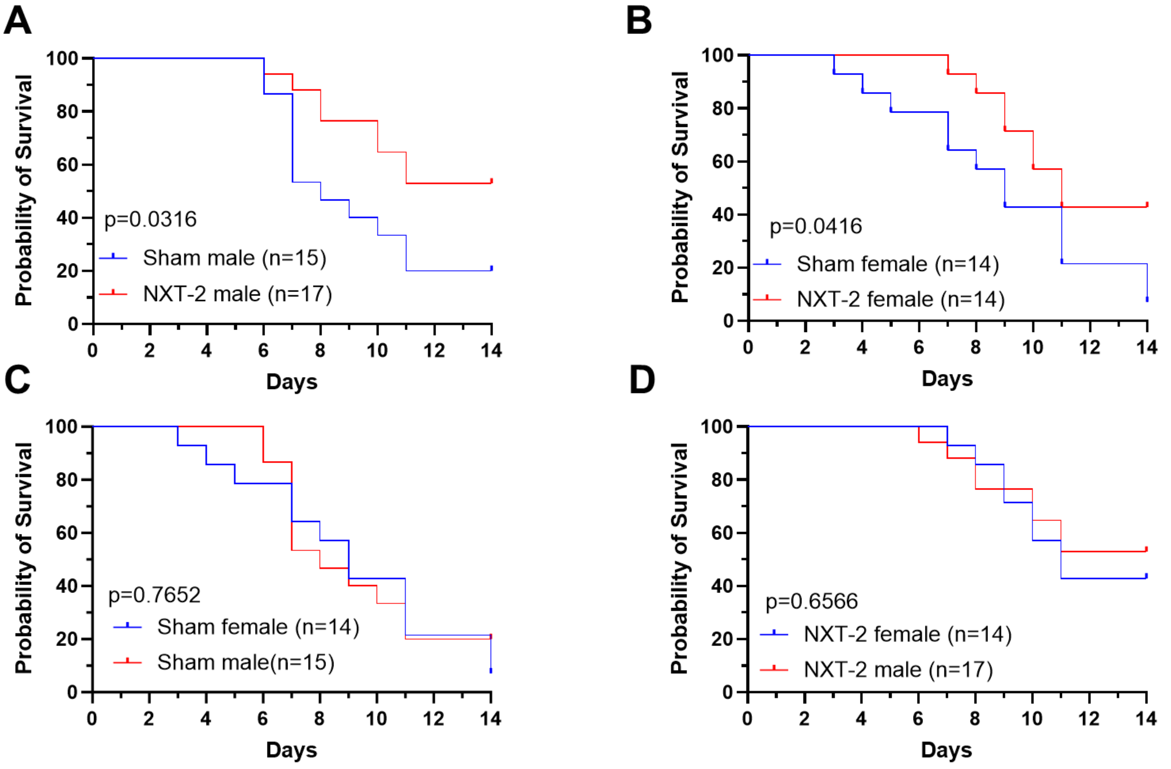

Supplementary Figure S1: **Survival of male and female mice after *C. auris* challenge**. Survival of mice immunized with NXT-2 was significantly increased compared to sham-immunized mice between **(A)** male mice (p=0.0316) and **(B)** female mice (p=0.0416). There was no statistical difference in survival between **(C)** sham-immunized mice (p= 0.7652) and **(D)** NXT-2 immunized mice (p= 0.6566)

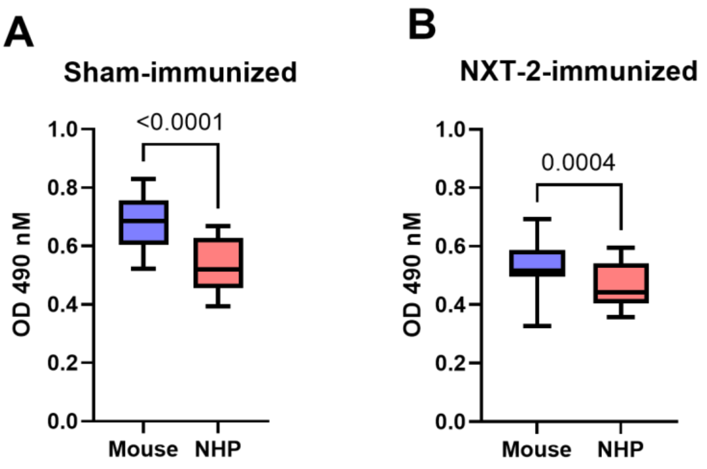

Supplementary Figure S2: **Comparison of biofilm formation using mouse and NHP sera**. C. auris biofilm inhibition is significantly higher in NHP sera compared to mouse sera for both **(A)** sham-immunized animals (p>0.0001) and **(B)** NXT-2-immunized animals (p=0.0004).

Full length blots

1. Fig. 1A CAu.KEX1 stained with Coomassie blue

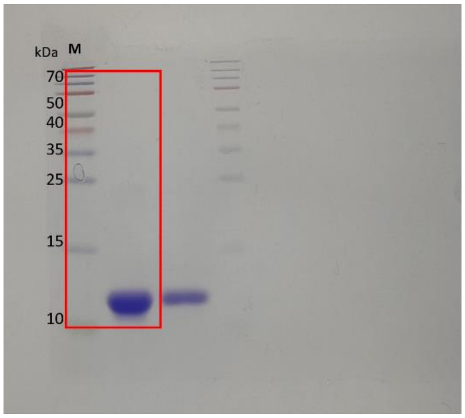

2. Fig. 1Bi Pre C. *auris* challenge

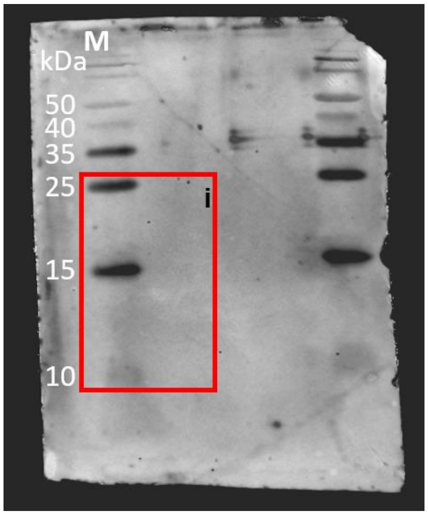

3. Fig 1Bii Post *C. auris* challenge

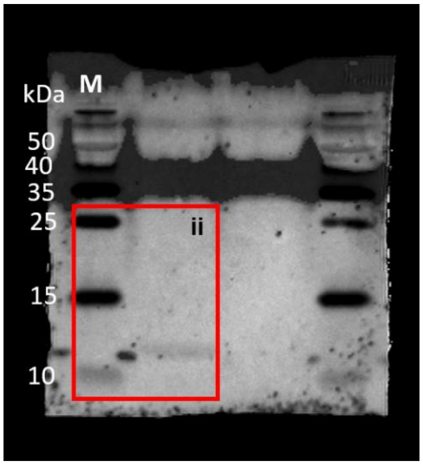

4. Fig 1Ci Sham-immunized

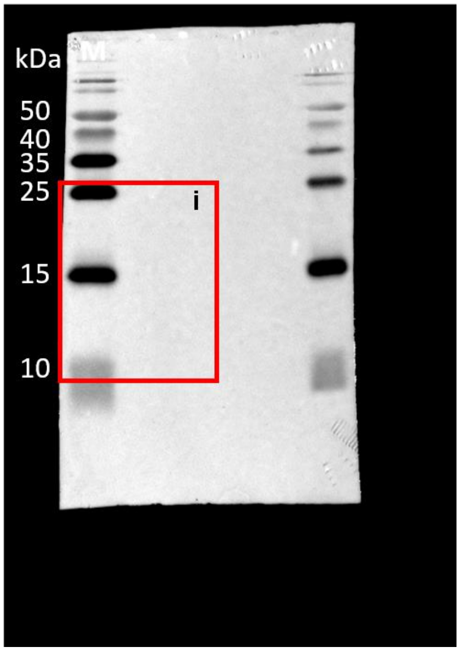

5. Fig 1Cii NXT-2 immunized

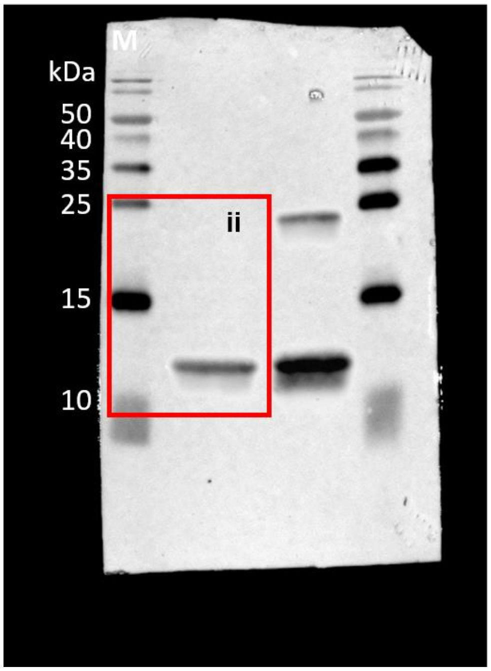

Supplement: Supplementary file 1 [file vaccines-13-01033-s001.zip › vaccines-3883687-supplementary.pdf]
